# Novel Targets and Therapeutic Strategies to Protect Against Hepatic Ischemia Reperfusion Injury

**DOI:** 10.3389/fmed.2021.757336

**Published:** 2022-01-04

**Authors:** Xin-li Mao, Yue Cai, Ya-hong Chen, Yi Wang, Xiu-xiu Jiang, Li-ping Ye, Shao-wei Li

**Affiliations:** ^1^Key Laboratory of Minimally Invasive Techniques and Rapid Rehabilitation of Digestive System Tumor of Zhejiang Province, Taizhou Hospital of Zhejiang Province Affiliated to Wenzhou Medical University, Linhai, China; ^2^Department of Gastroenterology, Taizhou Hospital of Zhejiang Province Affiliated to Wenzhou Medical University, Linhai, China; ^3^Institute of Digestive Disease, Taizhou Hospital of Zhejiang Province Affiliated to Wenzhou Medical University, Linhai, China; ^4^Health Management Center, Taizhou Hospital of Zhejiang Province Affiliated to Wenzhou Medical University, Linhai, China; ^5^Department of Urology, The Second Affiliated Hospital of Kunming Medical University, Kunming, China

**Keywords:** novel target, therapeutic strategy, ischemia reperfusion, injury, liver

## Abstract

Hepatic ischemia reperfusion injury (IRI), a fascinating topic that has drawn a lot of interest in the last few years, is a major complication caused by a variety of clinical situations, such as liver transplantation, severe trauma, vascular surgery, and hemorrhagic shock. The IRI process involves a series of complex events, including mitochondrial deenergization, metabolic acidosis, adenosine-5'-triphosphate depletion, Kupffer cell activation, calcium overload, oxidative stress, and the upregulation of pro-inflammatory cytokine signal transduction. A number of protective strategies have been reported to ameliorate IRI, including pharmacological therapy, ischemic pre-conditioning, ischemic post-conditioning, and machine reperfusion. However, most of these strategies are only at the stage of animal model research at present, and the potential mechanisms and exact therapeutic targets have yet to be clarified. IRI remains a main cause of postoperative liver dysfunction, often leading to postoperative morbidity or even mortality. Very recently, it was reported that the activation of peroxisome proliferator-activated receptor γ (PPARγ), a member of a superfamily of nuclear transcription factors activated by agonists, can attenuate IRI in the liver, and FAM3A has been confirmed to mediate the protective effect of PPARγ in hepatic IRI. In addition, non-coding RNAs, like LncRNAs and miRNAs, have also been reported to play a pivotal role in the liver IRI process. In this review, we presented an overview of the latest advances of treatment strategies and proposed potential mechanisms behind liver IRI. We also highlighted the role of several important molecules (PPARγ, FAM3A, and non-coding RNAs) in protecting against hepatic IRI. Only after achieving a comprehensive understanding of potential mechanisms and targets behind IRI can we effectively ameliorate IRI in the liver and achieve better therapeutic effects.

## Introduction

Good blood circulation is a known prerequisite for maintaining a normal organ function, which can be seriously compromised by ischemic disease. Ischemia reperfusion injury (IRI) is a phenomenon wherein organ cell damage caused by hypoxia is paradoxically aggravated after the restoration of blood circulation and oxygen delivery (1). This is a dynamic process involving two related stages: ischemic injury and inflammation-mediated reperfusion injury (2). Theoretically, IRI can occur in a variety of tissues and organs, such as the liver, heart, kidney, lung, intestine, and central nervous system. If severe enough, the local release of free radicals and inflammatory response after IRI can even spread throughout the whole body, leading to systemic inflammatory response syndrome (SIRS) or multiple organ dysfunction syndrome (MODS) (3). The liver, being the largest solid organ in the body, is highly dependent on an oxygen supply for its energy metabolism and is susceptible to hypoxia. Therefore, hepatic IRI is a frequent and major complication caused by diverse clinical situations, such as liver transplantation (LT), severe trauma, vascular surgery, and hemorrhagic shock (4).

In recent years, hepatic IRI has drawn substantial interest as a research topic. The process involves a series of complex events, including mitochondrial deenergization, metabolic acidosis, adenosine-5'-triphosphate depletion, Kupffer cell activation, calcium overload, oxidative stress, and the upregulation of pro-inflammatory cytokine signal transduction (5–8). Such diversity of liver IRI mechanisms has contributed to the pathophysiology of the disease, which has made it difficult to achieve effective protection by targeting a single mediator or mechanism to reduce IRI, either in clinical practice or experiments (9). A number of protective strategies have been reported to ameliorate IRI, including pharmacological therapy, ischemic pre-conditioning (IPC), ischemic post-conditioning (IPostC), and machine reperfusion (10–14). However, most of these strategies are only at the stage of animal model research at present, and the potential mechanisms and exact therapeutic targets have yet to be clarified. IRI remains one of the main cause of postoperative liver dysfunction, often leading to postoperative morbidity or even mortality. Very recently, it was reported that the activation of peroxisome proliferator-activated receptor γ (PPARγ), a member of a superfamily of nuclear transcription factors activated by agonists, can attenuate IRI in the liver (15, 16), and FAM3A has been confirmed to mediate the protective effect of PPARγ in hepatic IRI (17). In addition, non-coding RNAs, like LncRNAs and miRNAs, have also been reported to play a pivotal role in the process of liver IRI (18, 19).

In this review, we presented an overview of the latest advances of treatment strategies and proposed potential mechanisms behind liver IRI. We also highlighted the role of several important molecules (PPARγ, FAM3A, and non-coding RNAs) in protecting against hepatic IRI. Notably, oral administration of PPARγ agonists before liver surgery to activate the FAM3A pathway might help ameliorate hepatic IRI. Only after achieving a comprehensive understanding of potential mechanisms and targets behind IRI, we can effectively ameliorate IRI in the liver and achieve better therapeutic effects.

## Contemporary Strategies and Approaches for Hepatic IRI

**Figure 1:**
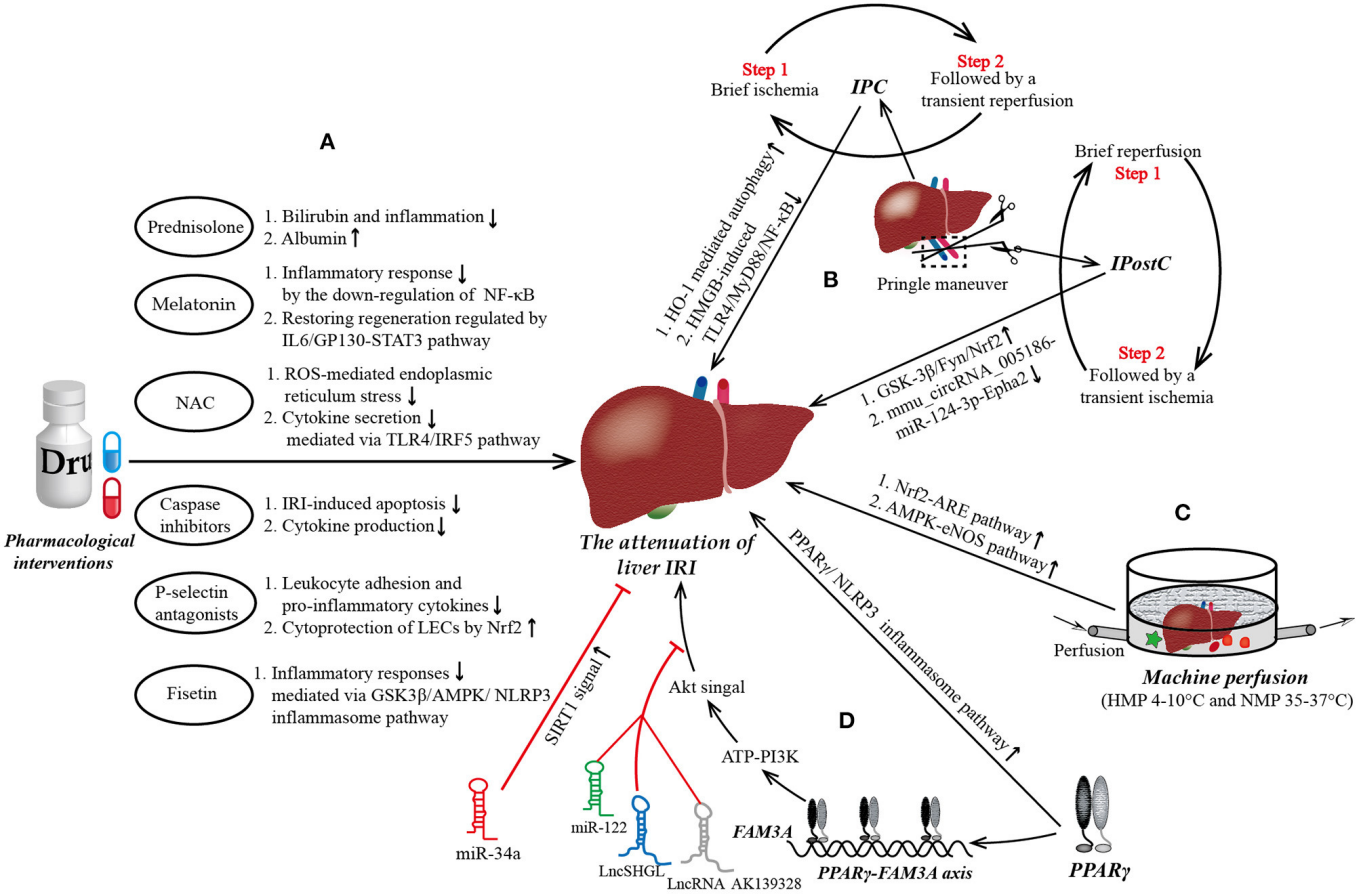
The mainstream strategies and novel targets to protect from liver ischemia reperfusion injury (IRI). **(A)** Pharmacological interventions in liver IRI. Most of current medicine used to attenuate liver IRI aim at combating the increased oxidative stress, reducing inflammatory response, inhibiting the apoptosis of hepatocytes, and promoting the regeneration of damaged liver tissue. **(B)** Ischemic preconditioning (IPC) and ischemic post-conditioning (IPostC) in liver IRI. Pringle maneuver (PM) is a simplest and most widely used technology of liver vascular clamping to achieve IPC prior to a prolonged period of ischemia and IPostC preceding a continuous reperfusion. IPC is characterized by a brief ischemia followed by a transient reperfusion, repeated several times, while the steps of IPostC are completely opposite. **(C)** Machine perfusion in liver IRI. Hypothermic machine perfusion (HMP) and normothermic machine perfusion (NMP) are the two main types in machine perfusion. Compared to HMP, NMP allows for the generation of data for the assessment of liver viability and allows for reconditioning of the liver *ex vivo* during the preservation period, which may allow the transplantation of currently deemed “untransplantable” organs in the near future. **(D)** Novel targets in liver IRI. Peroxisome proliferator-activated receptor γ (PPARγ) is a novel target to attenuate liver IRI maybe *via* ATP-PI3K-Akt pathway mediated by PPARγ-FAM3A axis. Of note, miR-122, LncRNA AK139328, and LncSHGL exert deleterious effects on liver IRI by blockading the signal transmission between Akt and the liver. NF-κB, nuclear factor kappa B; GP130, glycoprotein 130; STAT3, signal transducer and activator of transcription 3; ROS, reactive oxygen species; TLR4, Toll-like receptor 4; IRF5, interferon regulatory factor 5; LECs, liver endothelial cells; Nrf2, nuclear respiratory factor2; GSK3β, glycogen synthase kinase-3; AMPK, AMP activated protein kinase; NLRP3, domain-like receptor (NLR) family pyrin domain-containing 3; HMGB, high mobility group box; MyD88, myeloid differential protein-88; Epha2, ephrin type-A receptor 2; ARE, antioxidant response element; eNOS, endothelial nitric oxide synthase.

Mainstream approaches to preventing hepatic IRI have included various pharmacological interventions, IPC, IPostC, and machine perfusion (10–14). Multiple drugs have been used to prevent liver IRI, the main purposes of which are to combat the increased oxidative stress during liver IRI or to apply immunomodulation. Of note, variations in existing liver clamp techniques, designed to reduce IRI by manufacturing IPC and IPostC, have been extensively studied and are routinely implemented in liver transplantation and resection (Figure 1). We will now briefly review some of these approaches.

### Pharmacological Interventions

Since the pathophysiology of hepatic IRI involves multiple targets and mechanisms, various types of pharmacological interventions are currently being tested to suppress this phenomenon (Table 1). During liver surgery, methylprednisolone, trimetazidine, and ulinastatin reportedly help mitigate IRI and thereby render protection for the liver (10). However, the routine use of these drugs to reduce IR in controlled liver resection remains a controversial issue owing to the lack of sufficient clinical trials.

**Table 1:** Summarization of pharmacological interventions in liver IRI.

**Pharmacological ingredient**	**Data origin**	**Main effects and conclusion**	**References**
Prednisolone	Clinical trial	Glucocorticoid improves postoperative liver function and enhances the perioperative safety.	(20)
Prednisolone	Rat model	Liposomal encapsulation efficiently mediates local delivery of glucocorticoids.	(5)
Melatonin	Mouse model	Melatonin restore regeneration in IRI, perhaps *via* the IL6/GP130-STAT3 signal pathway.	(21)
Melatonin	Rat model	Melatonin ameliorates inflammation by inhibiting the activity of NF-κB signaling.	(22)
N-acetylcysteine (NAC)	Mouse model	NAC alleviates liver injury *via* inhibiting the TLR4/IRF5 signaling pathway.	(23)
N-acetylcysteine	Clinical trial	NAC reduces the probability of an abnormal liver function postoperatively.	(24)
P-selectin antagonists	Rat model	It blocks the migration of leukocytes into the IR-stressed liver and acts as a cytoprotective agent of LECs by Nrf2 signal.	(7)
P-selectin antagonists	Clinical trial	It blockades leukocyte adhesion and reduces the production of pro-inflammatory cytokines.	(25)
Caspase inhibitor	Mouse model	It reduces cellular injury, decreases apoptosis, and improves cytokine profiles.	(26)
Caspase inhibitor	Clinical trial	It inhibits IRI-induced apoptosis, delays graft dysfunction, and ameliorates hepatic injury.	(27)
Prostacyclin	Rat model	Iloprost reduces plasma liver transaminase levels and increases antioxidant enzymes levels.	(28)
Prostacyclin	Rat model	Treprostinil reduces hepatic necrosis, preserves the inner wall of sinusoidal endothelial cells, restored energy and reduced platelet deposition.	(29)
Prostacyclin	Clinical trial	Treprostinil decreases the occurrence of PNF, minimizes the need for ventilation support, and improves hepatobiliary excretion.	(30)
Nilotinib	Mouse model	Nilotinib decreases the expression of pro-inflammatory cytokines and the recruitment of inflammatory monocytes.	(31)
Fisetin	Mouse model	Fisetin counters inflammatory responses *via* the GSK3β/AMPK/NLRP3 inflammasome pathway.	(32)
Gastrodin	Mouse model	Gastrodin protects liver against IRI by activating the Nrf2/HO-1 pathway.	(33)

*GP130, glycoprotein 130; STAT3, signal transducer and activator of transcription 3; NF-κB, nuclear factor kappa B; LECs, liver endothelial cells; Nrf2, nuclear respiratory factor 2; PNF, primary graft non-function; GSK3β, glycogen synthase kinase-3; AMPK, AMP activated protein kinase; NLRP3, domain-like receptor (NLR) family pyrin domain-containing 3; HO-1, heme oxygenase-1*.

#### Prednisolone

Prednisolone is a glucocorticoid steroid that generally acts as an anti-inflammatory agent in experimental liver IRI, being able to reduce both inflammation markers and apoptotic cell counts (34). A systematic review and meta-analysis by Orci et al. (35) concerning the effects of perioperative steroids on IRI showed that perioperative steroids are associated with favorable postoperative outcomes after hepatectomy. The postoperative morbidity of patients receiving intravenous glucocorticoids was 24% lower than that of the control group. In addition, steroids can significantly reduce the level of blood bilirubin and inflammation markers postoperatively, such as interleukin 6 (IL-6) and C-reactive protein (CRP). Nevertheless, there was no evidence of significant differences between the two study groups in terms of infection and wound healing complications. Similarly, a double-blind, randomized controlled trial enrolling 124 cases recently conducted by Hasegawa et al. (20) revealed that the preoperative use of glucocorticoids in laparoscopic liver resection significantly improved the postoperative liver function. The levels of bilirubin, CRP, and IL-6 in the glucocorticoid group of that study were significantly lower while the albumin level was significantly higher and the prothrombin time significantly shorter than in the control group. Furthermore, to reduce the adverse effects of systemic high-dose hormones and increase the local hormone concentration, a recent study by van Alem et al. (5) showed that liposomal encapsulation could efficiently mediated local delivery of glucocorticoids in rats, suggesting its potential utility as a treatment strategy. Nevertheless, animal models constructed in this this study are renal IRI rats, and whether the role of liposomal encapsulation for local delivery of glucocorticoids is equally effective in liver IRI still needs further verification.

#### Melatonin

In addition to prednisolone, melatonin, a puissant endogenous antioxidant synthesized in the pineal gland, has also shown potential value in the treatment of liver IRI (36). Melatonin can directly act as a free radical scavenger or indirectly act by upregulating the expression of several antioxidant enzymes (superoxide dismutase, glutathione peroxidase, glutathione reductase, etc.) to alleviate liver IRI (37, 38). Importantly, a number of studies have shown that melatonin attenuates liver IRI by maintaining the stability of the mitochondrial membrane, ensuring synthesis of ATP in the liver, and improving bile production in rat models of cold storage (39–42). Furthermore, an animal experimental study published in J Pineal Res in 2018 (21) demonstrated that melatonin could attenuate liver graft IRI, restore regeneration, and improve the recipient survival outcome, perhaps *via* the IL6/GP130-STAT3 signal pathway. Exogenous melatonin was found to promote the release of IL-6, IL-10, and tumor necrosis factor-α (TNF-α) by infiltrating inflammatory Ly6C+ F4/80+ monocytes, the critical role of which were IL-6 secreted by monocytes and downstream GP130-STAT3 signaling. Another animal study by Chinese scholars Gao et al. (22) recently showed that melatonin was able to downregulate the activity of the nuclear factor kappa B (NF-κB) signaling pathway in the early and late stages of hepatic IRI, ameliorate the inflammatory response, protect the liver function, and improve the survival rate in the perioperative period. The authors randomly divided 60 Sprague-Dawley rats into a sham group, ischemia-reperfusion injury group (I/R group), and melatonin-treated group (M+ I/R group). Immunofluorescence staining showed that the expression of NK-κB in the M+ I/R group was significantly lower than that in the I/R group, and the 7-day survival rate in M+ I/R group was higher than that in the I/R group (50 vs. 20%, *p* < 0.05).

#### N-Acetylcysteine (NAC)

NAC, a glutathione precursor, has also been reported to protect the liver from IRI in animal models (6). Its protective effects against liver IRI may be exerted *via* the regulation of the ROS/JNK/Bcl-2 pathway to prevent IRI-induced autophagy and apoptosis (43) or by attenuating reactive-oxygen-species-mediated endoplasmic reticulum stress (44). Recently, an experimental study by Nasiri et al. (23) verified that Toll-like receptors (TLRs), especially TLR4, and their downstream factors, namely interferon regulatory factor 5 (IRF5), play a basic role in the inflammatory phase of IRI. The authors showed that pretreatment with NAC significantly inhibited the TLR4/IRF5 signaling pathway, thereby reducing the secretion of downstream cytokines after 3 h of reperfusion and subsequently alleviating liver injury after 168 h of reperfusion. A number of clinical trials have shown that intravenous injection of NAC can attenuate IRI in liver grafts, thereby reducing the occurrence of graft dysfunction and improving the liver function (45, 46). In 2016, a double-blind, randomized, control clinical trial containing 115 cases by Donadon et al. (24) showed that the intravenous administration of NAC prior to hepatic resection significantly reduced the probability of an abnormal liver function postoperatively compared with placebo when the Pringle maneuver exceeded 70 min. However, another clinical trial conversely found that the preservation of liver grafts inside University of Wisconsin solution plus NAC did not change the rate of IRI or influence the short-term prognosis (e.g., hospital stay, vascular complications, inotrope requirement, or in-hospital mortality) in liver transplant recipients (47). It was noteworthy that the application timing of NAC in the above two studies is different, in which one is to administer NAC intravenously before the liver is removed from the donor, and the other is to use NAC during the preservation of the donor liver. Therefore, the value of NAC in the prevention of liver IRI must be explored further, particularly *via* randomized and large-scale clinical trials to determine the translational value of NAC from the bench to the bedside.

#### Noble Gases

The protective value of noble gases for the *ex vivo* preservation of organs prior to transplant and IRI during the perioperative period has been extensively verified in numerous preclinical experiments and clinical trials, with reports concerning argon and xenon being the most common (48–51). In a rat spinal I/R experimental model by Liu et al. (52), the rats in the I/R group with xenon-delayed post-conditioning had higher neurological scores, more normal motor neurons, fewer apoptotic neurons, and significantly higher levels of Akt and extracellular signalregulated kinase (ERK) than those without xenon-delayed post-conditioning, suggesting that xenon attenuated spinal cord IRI by activating the Akt and ERK signaling pathways. However, current research concerning the protective effect of xenon in the context of liver IRI remains limited. A recent study by German scholars Schmitz et al. (53) reported that argon inhalation had a detrimental effect on rat liver regeneration after IRI, which manifested in increased levels of the pro-inflammatory cytokines IL-1β and IL-6. Consequently, further studies especially clinical trials, are urgently needed to clarify the role of noble gases in preventing liver IRI.

#### P-Selectin and Caspase Inhibitor

The administration of P-selectin antagonists and caspase inhibitors has also been proven to be effective against hepatic IRI (54–57). It is common knowledge that liver endothelial cells (LECs) are the principal type of liver non-parenchymal cell and deemed to play a central role in regulating the substance exchange between the sinusoidal blood flow and surrounding tissues as well as maintaining the immune and coagulation functions (25). To analyze the role of LECs in combating IRI, Zhang et al. (25) used a novel recombinant soluble form of tandem P-selectin glycoprotein ligand-immunoglobulin (TSGL-Ig) in a rat model of syngeneic orthotopic liver transplantation. They consequently found that TSGL-Ig not only acted as a competitive antagonist to block the migration of leukocytes into the IR-stressed liver but may also have acted as a cytoprotective agent of LECs mediated by nuclear respiratory factor 2 (Nrf2) signal. This study supported the role of the P-selectin signaling pathway in liver homeostasis, with a wide range of influence on tissue damage. Clinically, a double-blind phase II study designed by Busuttil et al. (7) indicated that recombinant P-selectin glycoprotein ligand IgG successfully blockaded leukocyte adhesion, reduced the production of pro-inflammatory cytokines, and protected the function of the liver allograft.

With regard to caspase inhibitor, F573, a pan-caspase inhibitor, was reported to attenuate liver IRI in an *in vivo* murine IRI model, given the significantly reduced levels of markers of cellular injury, decreased evidence of apoptosis, and improved cytokine profiles compared to the vehicle (26). Coincidently, a recent experimental study by Fagenson et al. (58) indicated that Caspase 1/Caspase 11 double gene knockout can attenuate the liver IRI in mice. These promising results from animal researches lay a reasonable foundation for the introduction of caspase inhibitor to normothermic *ex situ* liver perfusion with the goal of improving the quality of marginal grafts. In order to validate the actual clinical application value, a phase II, multi-center, randomized, double-blinded, and parallel group study by Baskin-Bey et al. (27) designed to evaluate the utility of another pan-caspase inhibitor IDN-6556 in IRI injury during liver transplantation. IDN-6556 administered in cold storage and flush solutions, was able to inhibit IRI-induced apoptosis, delay graft dysfunction, and ameliorate hepatic injury based on the serum alanine aminotransferase (ALT) or aspartate aminotransferase (AST) levels. However, an unanticipated phenomenon occurred as intravenous administration of IDN-6556 to recipients after transplantation gradually abrogated the salutary effects of including IDN-6556 in the storage solution in a dose-dependent manner, which might be attributed to a massive accumulation of neutrophils in the graft caused by high doses of IDN-6556. Therefore, the clinical application of caspase inhibitor still needs more research support in the future.

#### Prostacyclin

PGI2 is a secret ingredient in normothermic *ex vivo* liver perfusion, with the properties of inhibiting platelet aggregation, leukocyte activation and chemotaxis, and superoxide anion production, which exerts an anti-inflammatory effects and protects endothelial cells (59, 60). To evaluate the effects of prostacyclin on the hepatic IRI, Gedik et al. (28) used iloprost, a prostacyclin analog, in a rat IRI model and showed that iloprost pretreatment decreased the plasma ALT and AST levels and upregulated the expression of antioxidant enzymes, such as superoxide dismutase (SOD), catalase (CAT), and glutathione peroxidase (GSH). Besides, regular sinusoidal structures with normal morphology without no signs of congestion were observed in the IRI rats pretreated with iloprost, whereas swollen hepatocytes with marked vacuolization were observed in the IRI rats without iloprost. Similar findings were reported by Ghonem et al. (29) that treprostinil, another prostacyclin analog, could significantly reduce serum liver transaminases, neutrophil infiltration, hepatic necrosis, and mRNA levels of pro-inflammatory cytokines in rat orthotopic liver transplantation (OLT). And treprostiil preserved the inner wall of sinusoidal endothelial cells, restored energy levels and reduced platelet deposition early after transplantation, and maintained a blood flow similar to the normal level compared to placebo.

To examine the actual clinical effect of prostacyclin, early in 2000, a placebo controlled randomized clinical trial by Neumann et al. (61) showed that treatment with prostacyclin tended to lower peak AST levels and induced a significant decrease in the difference between mixed venous oxygen content and hepatic venous oxygen content (delta O_2_) after 24 and 48 h after reperfusion, which indicated an improvement of regional hepatic-splanchnic oxygenation. To our interest, recently, a prospective, single-center, non-randomized, interventional study was performed by Almazroo et al. (30) to evaluate the safety and preliminary efficacy of perioperative treprostinil in preventing IRI in adult OLT recipients. In this study, subjects showed good tolerance to the continuous infusion of treprostinil without occurrence of primary graft non-function, which achieved 100% graft and recipient survival, minimized need for ventilation support, shortened hospitalization time, and improved hepatobiliary excretion comparable to normal healthy adults. Overall, prostacyclin shows pretty application prospects in ameliorating liver IRI, however, larger, randomized, double-blind, and multicenter clinical study is warranted to further demonstrate the role of prostacyclin in hepatic IRI.

#### Other Novel Pharmacological Interventions

Other novel pharmacological interventions reported to mitigate liver IRI are equally worthy of our attention. It is well-known that p38 Mitogen-activated protein kinases (MAPKs) signaling and c-Jun n-terminal kinases (JNKs) mediate liver IRI through inflammatory factor expression and cell death, respectively. Nilotinib, an oral receptor tyrosine kinase inhibitor originally used for chronic myeloid leukemia, also reportedly have *in vitro* activity against JNK and p38 MAPK (31). To further examine therapeutic potential of nilotinib against hepatic IRI, an experimental study from Memorial Sloan-Kettering Cancer Center, New York published in J Hepatol (31) was performed and showed that nilotinib markedly decreased the recruitment of inflammatory monocytes, reduced the expression of pro-inflammatory cytokines (IL-1β, IL-6, TNF, etc.), and ameliorated hepatocellular apoptosis in liver IRI in a murine model, perhaps *via* blocking activation of p38 MAPK signaling and JNK. In addition to nilotinib, Chinese medical herb also play an increasingly pivotal role in the management of liver IRI. Notably, Fisetin, a Chinese herbal medicinal ingredient with anti-inflammatory, anti-aging, and anti-oxidative properties, was reported to have the capability of countering inflammatory responses and mitigating hepatic IRI by downregulating the GSK3β/AMPK/NLRP3 inflammasome pathway (32). Coincidently, another recent study by Chinese scholars Yuan et al. (33) demonstrated that gastrodin pretreatment was able to protect liver against IRI in a murine model by activating the Nrf2/HO-1 pathway in a dose-responsive manner, leading to the downregulation of cytokines (IL-6 and TNF). Taken together, these findings suggest a novel and promising strategy for minimizing the injury induced by ischemia and reperfusion during liver transplantation. However, whether it be nilotinib, fisetin, or gastrodin, there are still limited data of these drugs clinically applied to ameliorating liver IRI in human.

### IPC and IPostC

#### IPC

IPC is a manipulative exercise involving short-term ischemia, commonly induced by portal triad clamping, followed by a brief period of reperfusion before an expected prolonged period of ischemia to protect IRI (Table 2). The protective effect of IPC on IRI was initially discovered by Murry et al. (72) in a canine myocardial ischemia model (72). A large number of experimental animal studies have provided strong preclinical evidence indicating that IPC can significantly improve the survival of hepatocytes and reduce the severity of liver IRI (11, 65). In 2016, Liu et al. (73) developed an IRI rat model indicating that inhibiting the activity of heme oxygenase-1 (HO-1) could reduce the autophagy induced by IPC, increase the activity of calpain2, and weaken the protective effect of IPC against liver IRI. Additionally, the inhibition of calpain2 was able to repair liver autophagy defects after I/R and reduce mitochondrial dysfunction. In general, IPC ameliorates liver IRI and restores the mitochondrial function, perhaps *via* HO-1-mediated autophagy. Similar results were observed in another recent experimental study showing that combined pretreatment of ischemia and rapamycin prevented hepatic IRI, perhaps *via* the restoration of autophagy activation in aged mice (62).

**Table 2:** Summarization of IPC/IPostC in liver IRI.

**IPC or IPostC**	**Data origin**	**Main effects and conclusion**	**References**
IPC	Rat model	IPC ameliorates liver IRI and restores mitochondrial function maybe *via* HO-1-mediated autophagy	(52)
IPC	Mouse model	Combined IPC and rapamycin protects against hepatic IRI attributing to restored autophagy activation	(62)
IPC	Rat model	Pretreatment with a slight of oxidative stress can trigger cellular adaptation protects by maintaining mitochondrial function.	(8)
IPC	Mouse model	IPC alleviates liver IRI by decreasing TIM4 expression.	(63)
IPC	Mouse model	RIPC diminishes hepatic IRI mediated by K_ATP_ through inhibition of HMGB1 induced TLR4/MyD88/NF-κB signaling pathway.	(64)
IPC	Clinical trial	RIPC neither significantly improves liver transaminase level nor decreased the incidence of EAD and PNF.	(65)
IPC	Clinical trial	RIPC does not improve liver function in living donor hepatectomy.	(66)
IPostC	Rat model	IPostC protects against liver IRI possibly *via* the GSK-3ß/Fyn/Nrf2 pathway.	(67)
IPostC	Rat model	IPostC protects against IRI mediated by microRNA-183 by repressing the expression of Apaf-1.	(68)
IPostC	Mouse model	Mmu_circRNA_005186-miR-124-3p-Epha2 pathway may be the key axis for IPostC to attenuate hepatic IRI.	(69)
IPostC	Clinical trial	Remote IPostC does not exhibit any improvements and clinical benefits preoperatively.	(70)
IPostC	Clinical trial	IPostC does not influence postoperative AST peak values, however, a better tolerance to IRI are observed.	(71)

*HO-1, heme oxygenase-1; TIM4, T-cell immunoglobulin and mucin domain molecule-4; TLR4, Toll-like receptors; MyD88, myeloiddifferentiationfactor88; NF-κB, nuclear factor kappa B; EAD, early allograft dysfunction; PNF, primary graft non-function; GSK3β, glycogen synthase kinase-3; Nrf2, nuclear respiratory factor 2. Apaf-1, apoptotic protease activating factor-1*.

Importantly, related studies have suggested that IRI occurs as a consequence of irreversible mitochondrial injury, and IPC is an endogenous protective mechanism that protects the mitochondrial function and biological energy (8). A slight increase in oxidative stress during IPC can trigger cellular adaptation by reducing the activity of ATP synthase, thereby increasing the tolerance to mitochondrial permeability transition (MPT) and maintaining ATP levels during liver IRI (8). T-cell immunoglobulin and mucin domain molecule-4 (TIM4), a substance conserved between mice and humans and mainly expressed in mature dendritic cells and macrophages, was considered to be necessary for macrophage migration, phagocytosis, and activation in the pathological process of liver IRI, and blocking the TIM4 signaling pathway could alleviate liver IRI (74). To determine whether TIM4 participated the process of IPC protecting against liver IRI, Zhang et al. (63) designed an experimental study and measured the expression of TIM4 in the each group (sham mouse group, IRI mouse group, and IPC+IRI mouse group). The results showed that IRI significantly increased the protein expression of TIM4, whereas IPC decreased the expression of TIM4 induced by IRI, which indicated that inhibiting TIM4 expression maybe another mechanism and potential therapeutic strategy for IPC to minimize liver IRI. Furthermore, another recent experimental study by Koh et al. (64) suggested that remote ischemic preconditioning (RIPC) diminished hepatic IRI appeared to be mediated by mitochondrial ATP-sensitive potassium channel (KATP) through inhibition of HMGB1 induced TLR4/MyD88/NF-κB signaling pathway. Serum liver injury transaminase levels, hepatic expression of inflammatory cytokines, and apoptosis-associated genes were lower in IR mice treated with RIPC compared to the observations in untreated IR mice. And the protective effects of RIPC against liver IRI were amplified in mice administrated KATP activator diazoxide, and, inversely, that the protective effect were weakened in those administrated with KATP inhibitor glyburide.

Although the protective effect of IPC on liver IRI has been verified in multiple animal model studies, contradictory results have emerged in ongoing clinical trials. A randomized clinical trial of 208 cases by Chinese scholars Qi et al. (65) showed that RIPC neither significantly improved the levels of ALT and AST in donors and recipients nor decreased the incidence of early allograft dysfunction (EAD), primary non-function, and postoperative complications in liver recipients. Coincidently, another randomized clinical trial recently published in Ann Surg (66) reported that RIPC was not associated with any marked differences in postoperative transaminase levels and did not decrease the incidence of a delayed graft hepatic function among donors. Furthermore, no marked differences in the incidence of EAD or graft failure were found among liver recipients.

Another major problem is that the optimal protocol, particularly concerning the ischemic intervals, remains poorly understood. The current IPC implementation methods mainly involve 5 min of ischemia/10 min of reperfusion and 10 min of ischemia/10 min of reperfusion, but which of these two methods is better is unclear. To help answer these questions, Lin et al. (75) recently compared several pretreatment protocols in a rat IRI model to determine the optimal IPC protocol. As a result, they found an IPC implementation method with 5 min of ischemia/5 min reperfusion, repeated three times, provided the best protection against liver IRI. Given the above findings, the application of IPC at the beside in actual patients appears premature.

#### IPostC

IPostC is a novel approach initiated in recent years to minimize IRI during the perioperative period of liver surgery (Table 2). In contrast to IPC, IPostC is defined as several brief cycles of reperfusion and ischemia after prolonged ischemia, involving controlled reperfusion prior to continuous reperfusion. Initially described by Zhao et al. (12), it was proven to effectively confer protection against liver IRI in subsequent studies (9, 76, 77). Compared to IPC, IPostC is more clinically relevant due to the timing procedure, wherein the conditioning stimulus is applied after prolonged ischemia and prior to permanent reperfusion, as opposed to IPC, where the stimulus must be initiated before the ischemic insult; as the onset of ischemia cannot always be predicted, IPostC is more practical than IPC.

Regarding the potential mechanisms involved, there are many hypotheses that IPostC could slow oxygenation through administering bursts of controlled reperfusion, inhibit neutrophil accumulation, increase antioxidant activity, modulate the apoptotic cascade, and upregulate the cytoprotective properties of nitric oxide (NO) (9, 76, 77). In addition, previous studies have shown the protective effects of IPostC against liver injury induced by IR *via* the glycogen synthase kinase 3 beta (GSK-3β)/Fyn/Nrf2 signaling pathway (67). In 2017, an experimental study published in Antioxid Redox Signal first demonstrated that IPostC protects against IRI mediated by microRNA-183 by repressing the expression of apoptotic protease activating factor-1 (Apaf-1), which is an apoptosis-promoting factor (68). Moreover, Chinese scholars Zhang et al. (69) also reported that circular RNAs (circRNAs) are closely related to hepatic IRI and IPostC, and the comprehensive circRNA expression profile, namely mmu_circRNA_005186-miR-124-3p-Epha2 pathway, may be the key axis for IPostC to attenuate hepatic IRI in a mouse model.

Nonetheless, the results from clinical trials differ markedly from the promising results obtained from animal experiments in research on using IPostC to ameliorate IRI. For example, a randomized controlled trial by Kim et al. (70) reported that remote ischemic post-conditioning (RIPostC) did not induce any improvements in the postoperative graft function, and no other clinical benefits with respect to the length of hospital stay, complication rate, or short-term mortality rate were observed. In that study, RPostC was performed with 5 min reperfusion/5 min ischemia, repeated four times, and the graft function was assessed through evaluations of the serum levels of total bilirubin and liver enzymes as well as the prothrombin time. Another observational study from Italy (71) showed that IPostC did not influence the postoperative AST peak, cellular apoptosis, morbidity, or 1-year graft survival rate. However, histological findings showed that IPostC was able to trigger autophagy in periportal areas, suggesting that grafts receiving IPostC obtain a better tolerance to IRI. Notably, the operation of IPostC in this study comprised three cycles of 1-min arterial occlusion interspersed with 1-min reperfusion pauses (71). These previous findings suggest that the implementation of IPostC is not yet uniform, and clinical test indicators are single, which might be reasons why IPostC has no protective effect against IRI in clinical practice. To better clarify the clinical impact of this approach, it is urgent to optimize the IPostC protocol and deepen our molecular understanding through future studies.

### Machine Reperfusion

From the acquisition of donor livers to the transplantation, liver grafts have been in the stage of preservation and repair. So far, static cold storage (SCS) is still the most commonly used liver preservation method, for its advantages of simplicity and economy. However, the low temperature and hypoxia of SCS has been reported to damage LECs, leading to delayed recovery or even loss of function of liver graft (78). Besides, with the aging and marginalization of donors, and the increasing proportion of cadaveric liver donors, disadvantages of SCS may become more prominent. Compared with traditional SCS, mechanical perfusion is a novel subject of much attention in organ preservation, with some unparalleled superiorities, such as dynamic circulation to maintain normal physiological metabolism, data generation to assess liver graft viability, and *ex vivo* reconditioning (78). In this section, we aim to review the application progress of machine perfusion in the field of liver transplantation.

#### Hypothermic Machine Perfusion (HMP)

Machine perfusion has always been a topic of interest in the field of liver transplantation, and growing evidence has implied that it may be the most efficient technique for attenuating IRI when applied to extended criteria donor (ECD) and donor after circulatory death (DCD) livers. Compared to SCS, the main method used to preserve allograft liver clinically for the past three decades, HMP is a novel technology involving perfusate containing diverse metabolic substrates and other protective mediators that is circulated through the donor organ, thereby flushing cytokines and toxins from the organ. Another advantage of HMP is that it allows handlers to judge the acceptability of the allograft based on determinations of pump parameters and enzymes in the preservation solution (13) HMP is usually zeperformed between 4 and 10°C, although the optimum temperature remains controversial. Lower temperatures have the advantage of inhibiting metabolic activity but require a higher perfusion pressure, which increases the risk of sinusoidal endothelial cell (SEC) injury. A group from China showed that rat DCD liver preserved by HMP exhibited improved histological results, higher levels of ATP, and greater bile production and superoxide dismutase (SOD) activation than those preserved by cold storage, perhaps by activating the Nrf2-antioxidant response element (ARE) signaling pathway (79). Interestingly, the addition of metformin to HMP perfusate *in vitro* is reported to significantly relieve hepatic injury during cold ischemia by activating the AMPK-eNOS mediated pathway (80).

However, while studies concerning HMP in animal models of liver IRI have been promising, clinical trials of liver HMP remain in their infancy. A meta-analysis by Zhang et al. (81) incorporated 6 clinical trials of liver HDM, involving 178 and 144 liver allografts preserved by SCS and HMD, respectively. The incidences of biliary complications and EAD were found to be significantly decreased with the 1-year graft survival significantly greater in the HMP group than in the SCS controls, whereas no significant difference was observed in terms of vascular complications, the incidence of primary non-function (PNF), or the length of hospital stay between the two groups.

Notably, hypothermic oxygenation perfusion (HOPE) appears to be key to preventing IRI, the mechanism of which involves minimizing the initial release of mitochondrial reactive oxygen species (ROS) by reversibly downregulating the electron transfer rate. Lin et al. (82) added defatting cocktail to HOPE perfusate in a rat liver transplantation model, which was proven to be able to improve steatotic liver graft and postoperative survival compared to HOPE alone. The defatted liver transplantation can be safely used in liver transplantation in rats, which may lay a solid foundation for its subsequent clinical application. Another experimental animal study by Zhou et al. (83) indicated that HOPE alleviated DCD rat liver I/R by suppressing HECTD3-mediated TRAF3 polyubiquitination, which might be a useful target for improving IRI in DCD liver transplantation. To further investigate the clinical utility of HOPR for DCD liver, Switzerland scholars Dutkowski et al. (84) firstly performed a clinical trial involving eight patients with end stage liver diseases receiving DCD liver transplantation, and found that the application of HOPE for DCD livers appeared to be well-tolerated and easy-to-use, providing better clinical outcomes than the matched liver grafts from cardiac death donors.

However, two critical parameters for HOPE have yet to be clarified: the optimal perfusion route and the optimum level of oxygenation. Recently, a long-term efficacy study published in J Hepatol (85) reported that the use of HOPE in extended DCD livers had a similar 5-year survival to that of low-risk donor livers obtained after brain death in liver transplantation, suggesting that a simple end-ischemic perfusion approach may help widen the field for the safe utilization of DCD liver grafts.

#### Normothermic Machine Perfusion (NMP)

NMP is a novel method of organ preservation. It is special in that it uses blood-based perfusate to perfuse the liver and allows physiological metabolism. From the start of organ retrieval to when the organ is transplanted into the recipient, NMP can typically maintain the normal function of donor organs for 3–19 h (14). NMP have been reported to reduce the severity of liver IRI in numerous experimental studies, potentially by maintaining a physiological temperature and liver blood flow speed, regulating endothelial autophagy, and replenishing liver ATP storage (14, 86). NMP helps improve the prognosis of liver transplantation, and its utility has also been verified in clinical studies. A clinical trial from London published in Hepatology indicated that NMP successfully inhibited the pro-inflammatory response and promoted graft regeneration by altering gene-expression profiles in liver tissues. In that study, liver recipients receiving NMP grafts had significantly lower levels of AST than those receiving conventional cold storage (CS) grafts; furthermore, less necrosis and apoptosis in the parenchyma and lower neutrophil infiltration were also detected in NMP liver tissue than in CS liver tissues (87). However, while extending the preservation time of the liver graft can confer more opportunities for liver transplantation, the prolonged time afforded by NMP also brings new challenges, as red blood cells (RBCs) must combat with the prolonged sheer stress. To overcome this, another clinical trial by Laing et al. (88) present the first experience that acellular hemoglobin-based oxygen carrier (HBOC) Hemopure could act as an alternative oxygen carrier to packed RBCs in the NMP perfusion field. Besides, *in vitro* exposure to Hemopure was found to not change the level of ROS in the cells, nor did it induce an increase in apoptosis or necrosis in any of the tested cell lines.

Compared with HMP, NMP allows the generation of data for assessing the liver viability. Based on pre-clinical experiments, a comprehensive standard for evaluating the viability for transplantation based on the macroscopic appearance, lactic acid clearance, bile production, and vascular flow has been formed (89). These criteria were applied in a clinical trial by Mergental et al., in which six donor livers were declined by all UK transplant centers and subjected to NMP. Interesting, of these six donor livers, five met the viability criteria and were successfully transplanted, showing a normalized liver function within a month (90). Furthermore, Watson et al. (91) recommended a viability assessment of donor livers based on the bile pH and perfusate transaminase, as the liver's ability to produce alkaline bile is considered an acceptable indicator of the bile duct cell function. In the near future, metabolomics profiling and microRNA analyses may be competitive candidates in liver viability assessments (87, 92). Combined with these liver viability assessment protocols, NMP is expected to expand the liver source pool and improve the efficiency of the use of marginal liver transplantation.

Furthermore, another unique property of NMP compared with HMP is that it allows *ex vivo* reconditioning during the liver preservation period. The addition of defatting cocktails to the HOPE mentioned above also falls into this category (82). In addition, anti-inflammatory drugs can also be used to recondition the donor liver, which has been proven to significantly reduce the production of IL-6 and TNF-α and increase the level of IL-10 compared to vehicle (93). NMP allows for gene-based therapies, such as myr-Akt, which induces a cytoprotective effect against liver IRI (87). Adding mesenchymal stem cells (MSCs) to the perfusate allows for the improvement of the liver microcirculation and quality, perhaps by inhibiting macrophage activation and intercellular adhesion (94). NMP allows the reconditioning and modification of marginal livers, which will help ameliorate the shortage of livers for transplantation. Therefore, further research should focus on upgrading the NMP technical parameters and facilitating the transition from the bench to the beside.

### PPARγ and FAM3A in Liver IRI

PPARγ is a member of the PPAR subfamily, which is composed of PPARα, PPARβ/δ, and PPARγ. It is widely expressed in many tissues, including adipose tissue, skeletal muscle, and liver, controlling many intracellular metabolic processes as a ligand-induced nuclear receptor. Currently, synthetic agonists of PPARγ, such as pioglitazone and rosiglitazone are widely used to treat insulin-resistant diabetes. In recent years, PPARγ has also been confirmed to play a critical role in liver IRI. Its expression and activity in the liver after IRI are increased, which protects hepatocytes from necrosis and apoptosis (15). An experimental study by Xu et al. (16) showed that asiatic acid was effective in mitigating hepatic IRI through the attenuation of Kupper cell (KC) activation *via* the PPARγ/nucleotide-binding oligomerization domain-like receptor family pyrin domain-containing 3 (NLRP3) inflammasome signaling pathway (Figure 1). Similarly, Chinese scholars Liu et al. (95) reported that the activation of PPARγ by curcumin protected rats against liver IRI by modulating the polarization of KCs, suggesting its potential utility as therapy for IRI after liver transplantation. In addition, Ruan et al. (96) indicated that RIPC could reduce histologic damage and improve the liver function in rat hepatic IRI *via* the activation of PPAR-γ mediated autophagy. However, the target genes of PPARγ that directly mediate the protective effects against liver IRI remains poorly understood, hampering the clinical promotion of PPARγ agonists for attenuating liver IRI.

Recently, FAM3A, the first member of the sequence similarity 3 (FAM3) gene family, was identified as a target gene that directly docks with PPARγ. This proved that FAM3A is a new mitochondrial protein capable of promoting the synthesis and release of ATP (17). Chi et al. built an obese diabetic mice model and found that FAM3A could activate the ATP-P2 receptor-Akt pathway to induce the proliferation of 3T3L1 preadipocytes and vascular smooth muscle cells (VSMCs) (97). Furthermore, Akt has been proven to be an important survival kinase, which can phosphorylate some apoptotic molecules, including forkhead transcription factor, caspase 9, forkhead box protein O1 (FOXO1), and glycogen synthase kinase 3β (GSK-3β), beyond its well-known role in regulating lipid and sugar metabolism. To clarify the role and mechanism of FAM3A in liver IRI, an experimental study from China indicated that the expression of PPARγ and FAM3A in the mouse liver was found to be upregulated under IRI conditions, and the protective effects of PPARγ agonist liver IRI was *via* FAM3A-ATP-Akt pathway (98). Silencing FAM3A artificially would markedly exaggerate liver IRI, perhaps by inhibiting the production of ATP and the activity of Akt, thereby reducing the expression of anti-apoptotic genes and increasing the expression of pro-apoptotic genes in the liver (98). In addition to the Akt survival pathway, studies have demonstrated that NF-κB also play a key role in liver IRI by regulating the expression of pro-inflammatory cytokines in the liver (99). An experimental study by Chen et al. revealed that FAM3A inhibited the activity of NF-κB and inflammation in IRI rat livers. In that study, the authors pretreated animal models with rosiglitazone and were surprised to find that the activity of NF-κB and the expression of pro-inflammatory cytokines in the liver of mice were markedly suppressed; furthermore, the above changes were not observed in FAM3A knockout mice, which showed more severe oxidative stress in the circulation and liver when suffering liver IRI.

In summary, FAM3A protects the liver from IRI by activating the Akt survival pathway, inhibiting the activity of NF-κB, and reducing oxidative stress. Furthermore, the protective effect of PPARγ against liver IRI are also likely mediated through the activation of the FAM3A pathway. Oral administration of PPARγ agonists before liver surgery to activate the hepatic FAM3A pathway may be a promising therapeutic strategy to ameliorate liver IRI, although available data of PPARγ to attenuate liver IRI in human remain to be limited (Figure 1).

### miRNA and Liver IRI

Non-coding RNAs are divided into small non-coding RNAs (<200 bp) and long non-coding RNAs (LncRNAs, ≥200 bp) according to their number of base pairs (100). miRNAs are a type of non-coding RNA, usually comprised of 19-24 nucleotides, and are the most important gene-regulatory factors at the post-transcriptional level. Previous studies have reported that miRNAs are closely associated with a variety of diseases or dysfunction (18). Chinese scholars Ng et al. (19) reported that miRNAs may also be involved in liver IRI, as the expression of several circulating miRNAs, including miR-1246, miR-148a, and miR-1290, was upregulated after liver transplantation. Notably, the overall expression profile of miRNAs in the liver of mice differed significantly not only between the reperfusion sample and the sham control but also between the ischemia sample and sham control (101). In this part, we review the latest research concerning the role and mechanism of several important miRNAs in liver IRI.

miR-122 is the most abundant miRNA in the liver, accounting for almost 70% of the total amount of liver miRNA (18). In 2012, Andersson et al. designed an ischemic porcine cardiogenic shock model and found that circulating miR-122 levels were increased nearly 460,000-fold after cardiogenic shock, whereas classic markers of hepatocellular necrosis were only modestly elevated nearly 3-fold, and miR-122 levels significantly decreased after therapeutic hypothermia (102). Van Caster et al. (103) reported that the level of miR-122 increased in correlation with the serum activities of ALT, AST, and lactate dehydrogenase (LDH) in rats during the period of warm hepatic IRI. These experimental researches all suggested that miR-122 may act as a potential biomarker of warm hepatic IRI. To further elucidate the role of miR122 in liver IRI, Xiao et al. conducted a cell anoxia-reoxygenation injury model and found that mild hypothermia pretreatment attenuated liver IRI and down-regulated the expression of miR-122, but these protective effects were abrogated by overexpressed miR-122 blockade. And in-depth analysis indicated that down-regulation of miR-122 promoted insulin-like growth factor 1 receptor (IGF-1R) translation and AKT activity, and inhibited Caspase3 expression and FOXO3a activity (104) (Figure 1). Moreover, a comparative experimental model study by Mard et al. (105) noted that crocetin, ZnSO4, and the combination thereof prevented hepatic IRI in rats, *via* inhibiting the expression of miR-122, and increasing antioxidant activity and Nrf2 expression. There might a link between miR122 and Nrf2, but needed further research to confirm. Collectively, these findings suggest that miR-122 may be is a potential therapeutic targets to ameliorate liver IRI.

The mature miR-34 subfamily comprises three members: miR-34a, miR-34b, and miR-34c, with miR-34a showing the closest connection to IRI (106). Wang et al. (107) reported that miR-34a-5p inhibition was able to reduce the accumulation of reactive oxygen species and apoptosis induced by intestinal ischemia/reperfusion by activating sirtuin 1 (SIRT1) signaling (Figure 1). Kim et al. (108) revealed that carbon monoxide (CO) reduced the inflammatory responses and hepatocellular apoptosis in a mouse liver IRI model by downregulating the miR-34a expression and upregulating the SIRT1 expression. Collectively, these findings suggest that the miR-34a/SIRT1 pathway may be a promising target for hepatic IRI therapy.

miR-223, which was initially identified as a regulator of hematopoietic differentiation, has been shown to be associated with liver IRI. In 2009, a group from China showed that the expression of miR-223 in mouse liver increased after 75 min of ischemia followed by 120 min of reperfusion compared to vehicle. Furthermore, the upregulation of miR-223 expression was positively correlated with serum AST and ALT activities (109), of which the downstream target genes were predicted to be acyl-CoA synthetase long-chain family member 3, ephrin A1, and ras homologous gene family member B. A recent study by Schueller et al. (110) showed that the level of miR-223 expression in the liver and serum from mice with acute liver injury (ALI) as well as in liver samples from patients with ALI was upregulated, and its level was markedly related to the degree of liver injury and liver cell death. In contrast, Van Caster et al. (103) observed an increase in miR-223 levels during hepatic IRI, but there was no correlation between this level and the serum AST/ALT activities. Collectively, these findings suggest that increased miR-223 expression in the liver is highly associated with liver IRI, but the specific mechanism is unclear.

## LncRNAs and Liver IRI

LncRNA is the another major group of non-coding RNAs widely involved in physiological and pathophysiological processes through cis-tether, cis-targeting, trans-targeting, enhancers, or co-inhibitors regulating the gene expression in diverse cell types.

Evidence has recently been provided that LncRN may play a critical role in liver injury. Su et al. (111) reported that the LncRNA TUG1 expression was downregulated in mouse liver after cold storage. Of note, the overexpression of TUG1 attenuated inflammation and oxidative stress as well as apoptosis of hepatocytes induced by cold storage *in vivo* and *in vitro*, suggesting that TUG1 may be a therapeutic target for preventing cold-induced liver injury in liver transplantation. In 2015, Chinese scholars Chen et al. first clarified the expression profile of LncRNA in a mouse IRI model based on microarray technology (112). In that study, up to 64 lncRNA were upregulated, and 244 lncRNA were downregulated in the plasma of mice after IRI. These findings suggest that certain plasma lncRNA may become a potential biomarker for liver IRI.

To further explore the role and exact mechanisms underlying the effects of LncRNA in liver IRI, Chen et al. (113) established a mouse IRI model and found that the inhibition of liver AK139328 led to a decrease in plasma transaminase activity and the area of liver necrosis. Simultaneously, slicing AK139328 increased the survival signaling proteins, such as phosphorylated Akt, endothelial nitric oxide synthase (peNOS), and glycogen synthase kinase 3 (pGSK3), in rat hepatic IRI models. A recent study by Wang et al. (114) reported that LncSHGL recruited heterogeneous nuclear ribonucleoprotein A1 (hnRNPA1) to increase the translation efficiency of CALM mRNA, thereby increasing the level of calmodulin (CaM) protein, leading to the activation of the phosphatidylinositol 3-kinase (PI3K)/Akt pathway and finally suppressing hepatic gluconeogenesis and lipogenesis. Obviously, an increase in the expression of AK139328 plus a decrease in the expression of LncSHGL together exert a deleterious effect on hepatic IRI by impairing the activity of Akt signaling (Figure 1). In addition, Tang et al. (115) revealed that LncRNA HOTAIR regulated autophagy in hepatic IRI through the miR-20b-5p/ATG7 axis, potentially providing a novel target for the treatment of liver IRI. Another LncRNA called metastasis-associated lung adenocarcinoma transcript 1 (MALAT1) was reported to aggravate liver IRI, potentially by regulating the apoptosis and inflammation triggered by HMGB1-TLR4, suggesting that MALAT1 might be a promising treatment strategy against hepatic IRI (116).

## Conclusion

**Figure 2:**
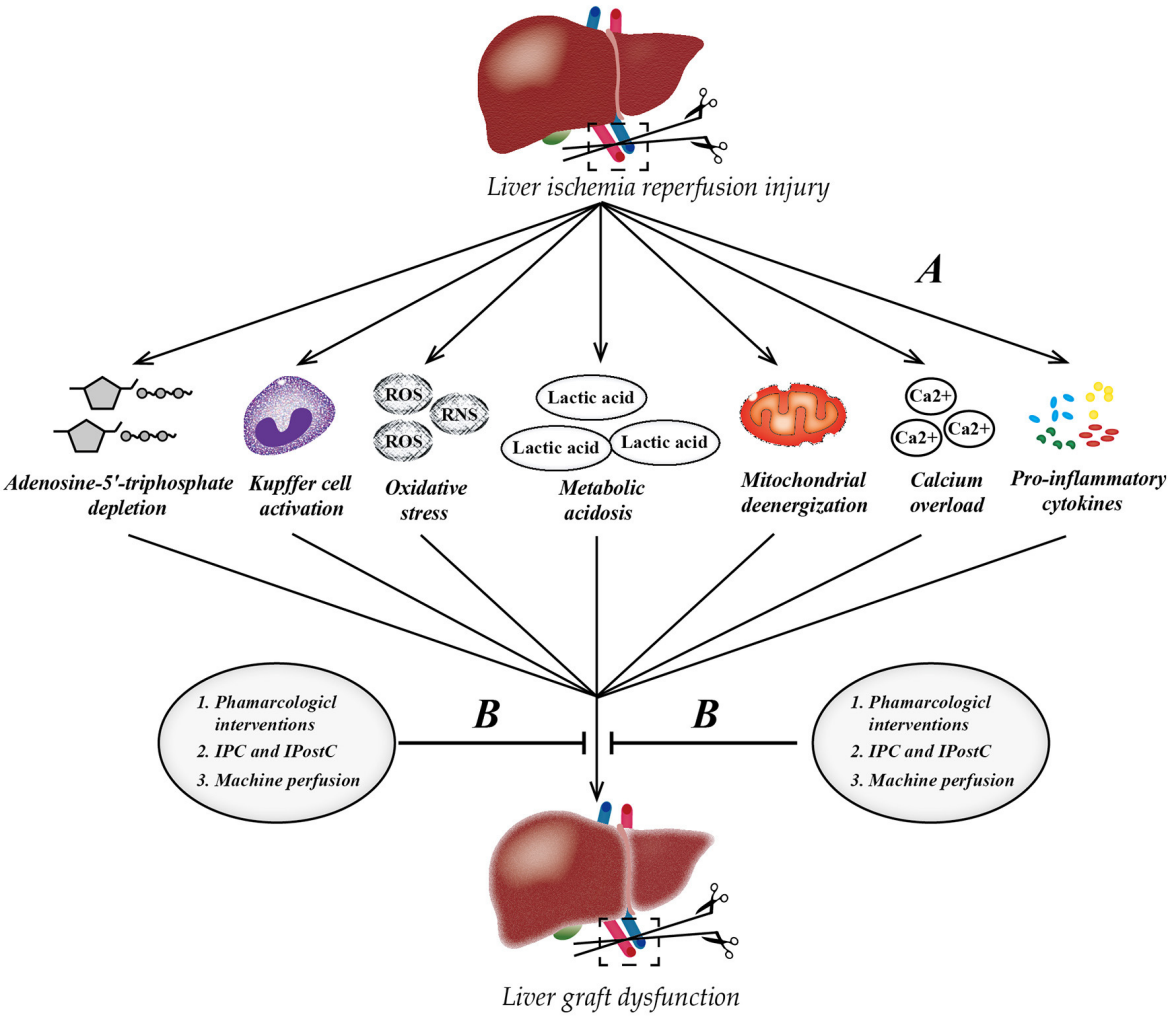
Pathophysiology of liver ischemia-reperfusion injury (IRI) and therapeutic strategies. **(A)** The process of IRI involves a series of complex events, such as mitochondrial deenergization, metabolic acidosis, adenosine-5'-triphosphate depletion, Kupffer cell activation, calcium overload, oxidative stress, and upregulation of pro-inflammatory cytokine signal transduction. **(B)** Currently, there are three mainstream therapeutic strategies to attenuate hepatic IRI. IPC, ischemic preconditioning; IPostC, ischemic post-conditioning; ROS, reactive oxygen species; RNS, reactive nitrogen species.

Hepatic IRI is a serious clinical problem that cannot be bypassed as the continuous promotion of liver transplantation, affecting millions of people worldwide. Pharmacological interventions (prednisolone, melatonin, n-acetylcysteine, noble gas, P-selectin inhibitor, caspase inhibitor, etc.), ischemia preconditioning, and post-ischemic preconditioning, as well as machine perfusion (HMP and NMP) may be effective strategies against liver IRI clinically. Of note, NMP allows the reconditioning of marginal livers, which will help ameliorate the shortage of livers for transplantation around the world (Figure 2). In the past decade, in-depth research has revealed the mechanism underlying liver IRI and identified many diagnostic biomarkers as potential therapeutic targets. Inhibiting certain miRNAs, such as miR-122 and miR-34a, regulating certain LncRNA, such as LncSHGL and HOTAIR, and activating the PPAR-FAM3A axis may be novel targets for treating liver IRI. Notably, the oral administration of PPARγ agonists before liver surgery to activate the FAM3A pathway might be a promising approach to ameliorating hepatic IRI. Only after achieving a comprehensive understanding of potential mechanisms and targets behind IRI can we effectively attenuate IRI in the liver and achieve better therapeutic effects.

## Author Contributions

All authors contributed to the writing and editing of the manuscript, contributed to the article, and approved the submitted version.

## Funding

This work was supported in part by Program of Technology Project of Zhejiang Province (2021PY083), Program of Taizhou Science and Technology Grant (20ywb29), Major Research Program of Taizhou Enze Medical Center Grant (19EZZDA2), Open Project Program of Key Laboratory of Minimally Invasive Techniques and Rapid Rehabilitation of Digestive System Tumor of Zhejiang Province (21SZDSYS01 and 21SZDSYS09), and Key Technology Research and Development Program of Zhejiang Province (2019C03040).

## Conflict of Interest

The authors declare that the research was conducted in the absence of any commercial or financial relationships that could be construed as a potential conflict of interest.

## Publisher's Note

All claims expressed in this article are solely those of the authors and do not necessarily represent those of their affiliated organizations, or those of the publisher, the editors and the reviewers. Any product that may be evaluated in this article, or claim that may be made by its manufacturer, is not guaranteed or endorsed by the publisher.

## References

[1] Zhou J Chen J Wei Q Saeb-Parsy K Xu X. The role of ischemia/reperfusion injury in early hepatic allograft dysfunction. Liver Transpl. (2020) 26:1034–48. 10.1002/lt.25779 32294292

[2] Nastos C Kalimeris K Papoutsidakis N Tasoulis MK Lykoudis PM Theodoraki K. Global consequences of liver ischemia/reperfusion injury. Oxid Med Cell Longev. (2014) 2014:906965. 10.1155/2014/906965 24799983 PMC3995148

[3] Black GE Sokol KK Moe DM Simmons JD Muscat D Pastukh V. Impact of a novel phosphoinositol-3 kinase inhibitor in preventing mitochondrial DNA damage and damage-associated molecular pattern accumulation: results from the Biochronicity Project. J Trauma Acute Care Surg. (2017) 83:683–9. 10.1097/TA.0000000000001593 28930961 PMC5938741

[4] Saidi RF Kenari SK. Liver ischemia/reperfusion injury: an overview. J Invest Surg. (2014) 27:366–79. 10.3109/08941939.2014.932473 25058854

[5] van Alem CMA Boonstra M Prins J Bezhaeva T van Essen MF Ruben JM. Local delivery of liposomal prednisolone leads to an anti-inflammatory profile in renal ischaemia-reperfusion injury in the rat. Nephrol Dial Transplant. (2018) 33:44–53. 10.1093/ndt/gfx204 28992069

[6] Hsieh CC Hsieh SC Chiu JH Wu YL. Protective effects of N-acetylcysteine and a prostaglandin E1 analog, alprostadil, against hepatic ischemia: reperfusion injury in rats. J Tradit Complement Med. (2014) 4:64–71. 10.4103/2225-4110.124351 24872935 PMC4032844

[7] Busuttil RW Lipshutz GS Kupiec-Weglinski JW Ponthieux S Gjertson DW Cheadle C. rPSGL-Ig for improvement of early liver allograft function: a double-blind, placebo-controlled, single-center phase II study. Am J Transplant. (2011) 11:786–97. 10.1111/j.1600-6143.2011.03441.x 21401865

[8] Teodoro JS Varela AT Duarte FV Gomes AP Palmeira CM Rolo AP. Indirubin and NAD(+) prevent mitochondrial ischaemia/reperfusion damage in fatty livers. Eur J Clin Invest. (2018) 48:e12932. 10.1111/eci.12932 29603199

[9] Toyoda T Tosaka S Tosaka R Maekawa T Cho S Eguchi S. Milrinone-induced postconditioning reduces hepatic ischemia-reperfusion injury in rats: the roles of phosphatidylinositol 3-kinase and nitric oxide. J Surg Res. (2014) 186:446–51. 10.1016/j.jss.2013.09.007 24120242

[10] Nemeth N Peto K Magyar Z Klarik Z Varga G Oltean M. Hemorheological and microcirculatory factors in liver ischemia-reperfusion injury-an update on pathophysiology, molecular mechanisms and protective strategies. Int J Mol Sci. (2021) 22:41864. 10.3390/ijms22041864 33668478 PMC7918617

[11] Yamada T Nagata H Kosugi S Suzuki T Morisaki H Kotake Y. Interaction between anesthetic conditioning and ischemic preconditioning on metabolic function after hepatic ischemia-reperfusion in rabbits. J Anesth. (2018) 32:599–607. 10.1007/s00540-018-2523-7 29931389

[12] Zhao ZQ Corvera JS Halkos ME Kerendi F Wang NP Guyton RA. Inhibition of myocardial injury by ischemic postconditioning during reperfusion: comparison with ischemic preconditioning. Am J Physiol Heart Circ Physiol. (2003) 285:H579–88. 10.1152/ajpheart.01064.2002 12860564

[13] Schlegel AA Kalisvaart M Muiesan P. Machine perfusion in liver transplantation: an essential treatment or just an expensive toy? Minerva Anestesiol. (2018) 84:236–45. 10.23736/S0375-9393.17.12016-X 28726360

[14] Boteon YL Laing R Mergental H Reynolds GM Mirza DF Afford SC. Mechanisms of autophagy activation in endothelial cell and their targeting during normothermic machine liver perfusion. World J Gastroenterol. (2017) 23:8443–51. 10.3748/wjg.v23.i48.8443 29358854 PMC5752706

[15] Marion-Letellier R Savoye G Ghosh S. Fatty acids, eicosanoids and PPAR gamma. Eur J Pharmacol. (2016) 785:44–9. 10.1016/j.ejphar.2015.11.004 26632493

[16] Xu Y Yao J Zou C Zhang H Zhang S Liu J. Asiatic acid protects against hepatic ischemia/reperfusion injury by inactivation of Kupffer cells *via* PPARgamma/NLRP3 inflammasome signaling pathway. Oncotarget. (2017) 8:86339–55. 10.18632/oncotarget.21151 29156799 PMC5689689

[17] Zhou Y Jia S Wang C Chen Z Chi Y Li J. FAM3A is a target gene of peroxisome proliferator-activated receptor gamma. Biochim Biophys Acta. (2013) 1830:4160–70. 10.1016/j.bbagen.2013.03.029 23562554

[18] Wang G Wan L Zhang L Yan C Zhang Y. MicroRNA-133a regulates the viability and differentiation fate of bone marrow mesenchymal stem cells *via* MAPK/ERK signaling pathway by targeting FGFR1. DNA Cell Biol. (2021) 40:1112–23. 10.1089/dna.2021.0206 34165368

[19] Ng KT Lo CM Wong N Li CX Qi X Liu XB. Early-phase circulating miRNAs predict tumor recurrence and survival of hepatocellular carcinoma patients after liver transplantation. Oncotarget. (2016) 7:19824–39. 10.18632/oncotarget.7627 26918346 PMC4991421

[20] Hasegawa Y Nitta H Takahara T Katagiri H Kanno S Umemura A. Glucocorticoid use and ischemia-reperfusion injury in laparoscopic liver resection: randomized controlled trial. Ann Gastroenterol Surg. (2020) 4:76–83. 10.1002/ags3.12298 32021961 PMC6992679

[21] Song Z Humar B Gupta A Maurizio E Borgeaud N Graf R. Exogenous melatonin protects small-for-size liver grafts by promoting monocyte infiltration and releases interleukin-6. J Pineal Res. (2018) 65:e12486. 10.1111/jpi.12486 29505662

[22] Gao Y Li ZT Jin L Lin J Fan ZL Zeng Z. Melatonin attenuates hepatic ischemia-reperfusion injury in rats by inhibiting NF-kappaB signaling pathway. Hepatobiliary Pancreat Dis Int. (2021). 4:1 10.1016/j.hbpd.2021.04.001 33947635

[23] Nasiri M Saadat M Karimi MH Azarpira N Saadat I. Evaluating mRNA expression levels of the TLR4/IRF5 signaling axis during hepatic ischemia-reperfusion injuries. Exp Clin Transplant. (2019) 17:648–52. 10.6002/ect.2017.0007 28969526

[24] Donadon M Molinari AF Corazzi F Rocchi L Zito P Cimino M. Pharmacological modulation of ischemic-reperfusion injury during Pringle maneuver in hepatic surgery. A prospective randomized pilot study. World J Surg. (2016) 40:2202–12. 10.1007/s00268-016-3506-1 27094558

[25] Zhang C Zhang Y Liu Y Liu Y Kageyama S Shen XD. A soluble form of P selectin glycoprotein ligand 1 requires signaling by nuclear factor erythroid 2-related factor 2 to protect liver transplant endothelial cells against ischemia-reperfusion injury. Am J Transplant. (2017) 17:1462–75. 10.1111/ajt.14159 27977895 PMC5444987

[26] Bral M Pawlick R Marfil-Garza B Dadheech N Hefler J Thiesen A. Pan-caspase inhibitor F573 mitigates liver ischemia reperfusion injury in a murine model. PLoS ONE. (2019) 14:e0224567. 10.1371/journal.pone.0224567 31770375 PMC6879152

[27] Baskin-Bey ES Washburn K Feng S Oltersdorf T Shapiro D Huyghe M. Clinical trial of the pan-caspase inhibitor, IDN-6556, in human liver preservation injury. Am J Transplant. (2007) 7:218–25. 10.1111/j.1600-6143.2006.01595.x 17227570

[28] Gedik E Girgin S Obay BD Ozturk H Ozturk H Buyukbayram H. Iloprost, a prostacyclin (PGI2) analogue, reduces liver injury in hepatic ischemia-reperfusion in rats. Acta Cir Bras. (2009) 24:226–32. 10.1590/S0102-86502009000300012 19504007

[29] Ghonem N Yoshida J Stolz DB Humar A Starzl TE Murase N. Treprostinil, a prostacyclin analog, ameliorates ischemia-reperfusion injury in rat orthotopic liver transplantation. Am J Transplant. (2011) 11:2508–16. 10.1111/j.1600-6143.2011.03568.x 21668631

[30] Almazroo OA Miah MK Pillai VC Shaik IH Xu R Dharmayan S. An evaluation of the safety and preliminary efficacy of peri- and post-operative treprostinil in preventing ischemia and reperfusion injury in adult orthotopic liver transplant recipients. Clin Transplant. (2021) 35:e14298. 10.1111/ctr.14298 33764591 PMC8243925

[31] Ocuin LM Zeng S Cavnar MJ Sorenson EC Bamboat ZM Greer JB. Nilotinib protects the murine liver from ischemia/reperfusion injury. J Hepatol. (2012) 57:766–73. 10.1016/j.jhep.2012.05.012 22641092 PMC3437237

[32] Pu JL Huang ZT Luo YH Mou T Li TT Li ZT. Fisetin mitigates hepatic ischemia-reperfusion injury by regulating GSK3beta/AMPK/NLRP3 inflammasome pathway. Hepatobiliary Pancreat Dis Int. (2021) 20:352–60. 10.1016/j.hbpd.2021.04.013 34024736

[33] Yuan B Huang H Qu S Zhang H Lin J Jin L. Gastrodin pretreatment protects liver against ischemia-reperfusion injury *via* activation of the Nrf2/HO-1 pathway. Am J Chin Med. (2020) 48:1159–78. 10.1142/S0192415X20500573 32668973

[34] Saidi RF Chang J Verb S Brooks S Nalbantoglu I Adsay V. The effect of methylprednisolone on warm ischemia-reperfusion injury in the liver. Am J Surg. (2007) 193:345–7. 10.1016/j.amjsurg.2006.09.017 17320532

[35] Orci LA Toso C Mentha G Morel P Majno PE. Systematic review and meta-analysis of the effect of perioperative steroids on ischaemia-reperfusion injury and surgical stress response in patients undergoing liver resection. Br J Surg. (2013) 100:600–9. 10.1002/bjs.9035 23339056

[36] Li Y Yang Y Feng Y Yan J Fan C Jiang S. A review of melatonin in hepatic ischemia/reperfusion injury and clinical liver disease. Ann Med. (2014) 46:503–11. 10.3109/07853890.2014.934275 25033992

[37] Dominguez-Rodriguez A Abreu-Gonzalez P Baez-Ferrer N Reiter RJ Avanzas P Hernandez-Vaquero D. Melatonin and cardioprotection in humans: a systematic review and meta-analysis of randomized controlled trials. Front Cardiovasc Med. (2021) 8:635083. 10.3389/fcvm.2021.635083 34055929 PMC8149621

[38] Mortezaee K Khanlarkhani N. Melatonin application in targeting oxidative-induced liver injuries: a review. J Cell Physiol. (2018) 233:4015–32. 10.1002/jcp.26209 29023783

[39] Kireev R Bitoun S Cuesta S Tejerina A Ibarrola C Moreno E. Melatonin treatment protects liver of Zucker rats after ischemia/reperfusion by diminishing oxidative stress and apoptosis. Eur J Pharmacol. (2013) 701:185–93. 10.1016/j.ejphar.2012.11.038 23220161

[40] Vairetti M Ferrigno A Bertone R Rizzo V Richelmi P Berte F. Exogenous melatonin enhances bile flow and ATP levels after cold storage and reperfusion in rat liver: implications for liver transplantation. J Pineal Res. (2005) 38:223–30. 10.1111/j.1600-079X.2004.00193.x 15813898

[41] De Deken J Rex S Monbaliu D Pirenne J Jochmans I. The efficacy of noble gases in the attenuation of ischemia reperfusion injury: a systematic review and meta-analyses. Crit Care Med. (2016) 44:e886–96. 10.1097/CCM.0000000000001717 27071065

[42] Ma Z Xin Z Di W Yan X Li X Reiter RJ. Melatonin and mitochondrial function during ischemia/reperfusion injury. Cell Mol Life Sci. (2017) 74:3989–98. 10.1007/s00018-017-2618-6 28795196 PMC11107672

[43] Wang C Chen K Xia Y Dai W Wang F Shen M. N-acetylcysteine attenuates ischemia-reperfusion-induced apoptosis and autophagy in mouse liver *via* regulation of the ROS/JNK/Bcl-2 pathway. PLoS ONE. (2014) 9:e108855. 10.1371/journal.pone.0108855 25264893 PMC4181678

[44] Kazaz IO Demir S Yulug E Colak F Bodur A Yaman SO. N-acetylcysteine protects testicular tissue against ischemia/reperfusion injury *via* inhibiting endoplasmic reticulum stress and apoptosis. J Pediatr Urol. (2019) 15:253 e1–8. 10.1016/j.jpurol.2019.02.005 30890312

[45] Bucuvalas JC Ryckman FC Krug S Alonso MH Balistreri WF Kotagal U. Effect of treatment with prostaglandin E1 and N-acetylcysteine on pediatric liver transplant recipients: a single-center study. Pediatr Transplant. (2001) 5:274–8. 10.1034/j.1399-3046.2001.005004274.x 11472606

[46] Bromley PN Cottam SJ Hilmi I Tan KC Heaton N Ginsburg R. Effects of intraoperative N-acetylcysteine in orthotopic liver transplantation. Br J Anaesth. (1995) 75:352–4. 10.1093/bja/75.3.352 7547057

[47] Aliakbarian M Nikeghbalian S Ghaffaripour S Bahreini A Shafiee M Rashidi M. Effects of N-acetylcysteine addition to university of Wisconsin solution on the rate of ischemia-reperfusion injury in adult orthotopic liver transplant. Exp Clin Transplant. (2017) 15:432–6. 10.6002/ect.2014.0263 26114393

[48] Smit KF Oei G Konkel M Augustijn QJJ Hollmann MW Preckel B. Plasma from volunteers breathing helium reduces hypoxia-induced cell damage in human endothelial cells-mechanisms of remote protection against hypoxia by helium. Cardiovasc Drugs Ther. (2019) 33:297–306. 10.1007/s10557-019-06880-2 31025141 PMC6538579

[49] Qi H Soto-Gonzalez L Krychtiuk KA Ruhittel S Kaun C Speidl WS. Pretreatment with argon protects human cardiac myocyte-like progenitor cells from oxygen glucose deprivation-induced cell death by activation of AKT and differential regulation of mapkinases. Shock. (2018) 49:556–63. 10.1097/SHK.0000000000000998 29658909

[50] Stevanovic A Schaefer P Coburn M Rossaint R Stoppe C Boor P. Renal function following xenon anesthesia for partial nephrectomy-an explorative analysis of a randomized controlled study. PLoS ONE. (2017) 12:e0181022. 10.1371/journal.pone.0181022 28719609 PMC5515428

[51] Smith SF Adams T Hosgood SA Nicholson ML. The administration of argon during *ex vivo* normothermic perfusion in an experimental model of kidney ischemia-reperfusion injury. J Surg Res. (2017) 218:202–8. 10.1016/j.jss.2017.05.041 28985850

[52] Liu S Yang Y Jin M Hou S Dong X Lu J. Xenon-delayed postconditioning attenuates spinal cord ischemia/reperfusion injury through activation AKT and ERK signaling pathways in rats. J Neurol Sci. (2016) 368:277–84. 10.1016/j.jns.2016.07.009 27538649

[53] Schmitz SM Dohmeier H Stoppe C Alizai PH Schipper S Neumann UP. Inhaled argon impedes hepatic regeneration after ischemia/reperfusion injury in rats. Int J Mol Sci. (2020) 21:155457. 10.3390/ijms21155457 32751707 PMC7432339

[54] Fusco R Cordaro M Siracusa R Peritore AF Gugliandolo E Genovese T. Consumption of *Anacardium occidentale* L. (cashew nuts) inhibits oxidative stress through modulation of the Nrf2/HO-1 and NF-kB pathways. Molecules. (2020) 25:194426. 10.3390/molecules25194426 32993187 PMC7582295

[55] Jones RT Toledo-Pereyra LH Quesnelle KM. Selectins in liver ischemia and reperfusion injury. J Invest Surg. (2015) 28:292–300. 10.3109/08941939.2015.1056920 26374984

[56] Kolachala VL Lopez C Shen M Shayakhmetov D Gupta NA. Ischemia reperfusion injury induces pyroptosis and mediates injury in steatotic liver thorough Caspase 1 activation. Apoptosis. (2021) 26:361–70. 10.1007/s10495-021-01673-1 33990906

[57] Telek V Erlitz L Caleb I Nagy T Vecsernyes M Balogh B. Effect of Pioglitazone on endoplasmic reticulum stress regarding *in situ* perfusion rat model. Clin Hemorheol Microcirc. (2021) 2021:211163. 10.3233/CH-211163 33867357

[58] Fagenson AM Xu K Saaoud F Nanayakkara G Jhala NC Liu L. Liver ischemia reperfusion injury, enhanced by trained immunity, is attenuated in caspase 1/caspase 11 double gene knockout mice. Pathogens. (2020) 9:110879. 10.3390/pathogens9110879 33114395 PMC7692674

[59] Echeverri J Goldaracena N Kaths JM Linares I Roizales R Kollmann D. Comparison of BQ123, epoprostenol, and verapamil as vasodilators during normothermic *ex vivo* liver machine perfusion. Transplantation. (2018) 102:601–8. 10.1097/TP.0000000000002021 29189484

[60] Zardi EM Dobrina A Amoroso A Afeltra A. Prostacyclin in liver disease: a potential therapeutic option. Expert Opin Biol Ther. (2007) 7:785–90. 10.1517/14712598.7.6.785 17555364

[61] Neumann UP Kaisers U Langrehr JM Glanemann M Muller AR Lang M. Administration of prostacyclin after liver transplantation: a placebo controlled randomized trial. Clin Transplant. (2000) 14:70–4. 10.1034/j.1399-0012.2000.140113.x 10693639

[62] Jiang T Zhan F Rao Z Pan X Zhong W Sun Y. Combined ischemic and rapamycin preconditioning alleviated liver ischemia and reperfusion injury by restoring autophagy in aged mice. Int Immunopharmacol. (2019) 74:105711. 10.1016/j.intimp.2019.105711 31302450

[63] Zhang Y Shen Q Liu Y Chen H Zheng X Xie S. Hepatic ischemic preconditioning alleviates ischemia-reperfusion injury by decreasing TIM4 expression. Int J Biol Sci. (2018) 14:1186–95. 10.7150/ijbs.24898 30123068 PMC6097479

[64] Koh WU Kim J Lee J Song GW Hwang GS Tak E. Remote ischemic preconditioning and diazoxide protect from hepatic ischemic reperfusion injury by inhibiting HMGB1-induced TLR4/MyD88/NF-kappaB signaling. Int J Mol Sci. (2019) 20:235899. 10.3390/ijms20235899 31771292 PMC6929132

[65] Qi B Wang XQ Pan ST Li PY Chen LK Xia Q. Effect of remote ischemic preconditioning among donors and recipients following pediatric liver transplantation: a randomized clinical trial. World J Gastroenterol. (2021) 27:345–57. 10.3748/wjg.v27.i4.345 33584067 PMC7852587

[66] Jung KW Kang J Kwon HM Moon YJ Jun IG Song JG. Effect of remote ischemic preconditioning conducted in living liver donors on postoperative liver function in donors and recipients following liver transplantation: a randomized clinical trial. Ann Surg. (2020) 271:646–53. 10.1097/SLA.0000000000003498 31356262

[67] Niu Q Sun W Chen Q Long Y Cao W Wen S. Protective effects of ischemic postconditioning on livers in rats with limb ischemia-reperfusion *via* glycogen synthase kinase 3 beta (GSK-3beta)/Fyn/Nuclear receptor-erythroid-2-related factor (Nrf2) pathway. Med Sci Monit. (2020) 26:e923049. 10.12659/MSM.923049 32686659 PMC7392060

[68] Lin HC Liu SY Yen EY Li TK Lai IR. microRNA-183 mediates protective postconditioning of the liver by repressing Apaf-1. Antioxid Redox Signal. (2017) 26:583–97. 10.1089/ars.2016.6679 27580417

[69] Zhang P Ming Y Ye Q Niu Y. Comprehensive circRNA expression profile during ischemic postconditioning attenuating hepatic ischemia/reperfusion injury. Sci Rep. (2019) 9:264. 10.1038/s41598-018-36443-8 30670716 PMC6342922

[70] Kim WH Lee JH Ko JS Min JJ Gwak MS Kim GS. Effect of remote ischemic postconditioning on patients undergoing living donor liver transplantation. Liver Transpl. (2014) 20:1383–92. 10.1002/lt.23960 25046844

[71] Ricca L Lemoine A Cauchy F Hamelin J Sebagh M Esposti DD. Ischemic postconditioning of the liver graft in adult liver transplantation. Transplantation. (2015) 99:1633–43. 10.1097/TP.0000000000000685 25856406

[72] Murry CE Jennings RB Reimer KA. Preconditioning with ischemia: a delay of lethal cell injury in ischemic myocardium. Circulation. (1986) 74:1124–36. 10.1161/01.CIR.74.5.1124 3769170

[73] Liu A Guo E Yang J Li R Yang Y Liu S. Ischemic preconditioning attenuates ischemia/reperfusion injury in rat steatotic liver: role of heme oxygenase-1-mediated autophagy. Oncotarget. (2016) 7:78372–86. 10.18632/oncotarget.13281 27852058 PMC5346646

[74] Ji H Liu Y Zhang Y Shen XD Gao F Busuttil RW. T-cell immunoglobulin and mucin domain 4 (TIM-4) signaling in innate immune-mediated liver ischemia-reperfusion injury. Hepatology. (2014) 60:2052–64. 10.1002/hep.27334 25066922 PMC4396987

[75] Lin J Huang H Yang S Duan J Xu W Zeng Z. Protective effects of ischemic preconditioning protocols on ischemia-reperfusion injury in rat liver. J Invest Surg. (2020) 33:876–83. 10.1080/08941939.2018.1556753 30821527

[76] Costa FL Teixeira RK Yamaki VN Valente AL Silva AM Brito MV. Remote ischemic conditioning temporarily improves antioxidant defense. J Surg Res. (2016) 200:105–9. 10.1016/j.jss.2015.07.031 26316445

[77] Chu WW He XY Yan AL Wang SW Li S Nian S. Ischemic postconditioning lightening ischemia/reperfusion apoptosis of rats *via* mitochondria pathway. Eur Rev Med Pharmacol Sci. (2019) 23:6307–14. 10.26355/eurrev_201907_18453 31364137

[78] Jia J Nie Y Li J Xie H Zhou L Yu J. A systematic review and meta-analysis of machine perfusion vs. static cold storage of liver allografts on liver transplantation outcomes: the future direction of graft preservation. Front Med. (2020) 7:135. 10.3389/fmed.2020.00135 32528963 PMC7247831

[79] Xue S He W Zeng X Tang Z Feng S Zhong Z. Hypothermic machine perfusion attenuates ischemia/reperfusion injury against rat livers donated after cardiac death by activating the Keap1/Nrf2ARE signaling pathway. Mol Med Rep. (2018) 18:815–26. 10.3892/mmr.2018.9065 29845199 PMC6059711

[80] Chai YC Dang GX He HQ Shi JH Zhang HK Zhang RT. Hypothermic machine perfusion with metformin-University of Wisconsin solution for *ex vivo* preservation of standard and marginal liver grafts in a rat model. World J Gastroenterol. (2017) 23:7221–31. 10.3748/wjg.v23.i40.7221 29142469 PMC5677206

[81] Zhang Y Zhang Y Zhang M Ma Z Wu S. Hypothermic machine perfusion reduces the incidences of early allograft dysfunction and biliary complications and improves 1-year graft survival after human liver transplantation: a meta-analysis. Medicine. (2019) 98:e16033. 10.1097/MD.0000000000016033 31169745 PMC6571373

[82] Lin F Zhen F Yan X Shaojun Y Guizhu P Yanfeng W. Hypothermic oxygenated perfusion with defatting cocktail further improves steatotic liver grafts in a transplantation rat model. Artif Organs. (2021) 45:E304–16. 10.1111/aor.13976 33908066

[83] Zhou W Zhong Z Lin D Liu Z Zhang Q Xia H. Hypothermic oxygenated perfusion inhibits HECTD3-mediated TRAF3 polyubiquitination to alleviate DCD liver ischemia-reperfusion injury. Cell Death Dis. (2021) 12:211. 10.1038/s41419-021-03493-2 33627626 PMC7904838

[84] Dutkowski P Schlegel A de Oliveira M Mullhaupt B Neff F Clavien PA. HOPE for human liver grafts obtained from donors after cardiac death. J Hepatol. (2014) 60:765–72. 10.1016/j.jhep.2013.11.023 24295869

[85] Schlegel A Muller X Kalisvaart M Muellhaupt B Perera M Isaac JR. Outcomes of DCD liver transplantation using organs treated by hypothermic oxygenated perfusion before implantation. J Hepatol. (2019) 70:50–7. 10.1016/j.jhep.2018.10.005 30342115

[86] Op den Dries S Karimian N Westerkamp AC Sutton ME Kuipers M Wiersema-Buist J. Normothermic machine perfusion reduces bile duct injury and improves biliary epithelial function in rat donor livers. Liver Transpl. (2016) 22:994–1005. 10.1002/lt.24436 26946466

[87] Jassem W Xystrakis E Ghnewa YG Yuksel M Pop O Martinez-Llordella M. Normothermic machine perfusion (NMP) inhibits proinflammatory responses in the liver and promotes regeneration. Hepatology. (2019) 70:682–95. 10.1002/hep.30475 30561835

[88] Laing RW Bhogal RH Wallace L Boteon Y Neil DAH Smith A. The use of an acellular oxygen carrier in a human liver model of normothermic machine perfusion. Transplantation. (2017) 101:2746–56. 10.1097/TP.0000000000001821 28520579 PMC5656179

[89] Perera T Mergental H Stephenson B Roll GR Cilliers H Liang R. First human liver transplantation using a marginal allograft resuscitated by normothermic machine perfusion. Liver Transpl. (2016) 22:120–4. 10.1002/lt.24369 26566737

[90] Mergental H Perera MT Laing RW Muiesan P Isaac JR Smith A. Transplantation of declined liver allografts following normothermic *ex-situ* evaluation. Am J Transplant. (2016) 16:3235–45. 10.1111/ajt.13875 27192971

[91] Watson CJE Kosmoliaptsis V Randle LV Gimson AE Brais R Klinck JR. Normothermic perfusion in the assessment and preservation of declined livers before transplantation: hyperoxia and vasoplegia-important lessons from the first 12 cases. Transplantation. (2017) 101:1084–98. 10.1097/TP.0000000000001661 28437389 PMC5642347

[92] Bruinsma BG Sridharan GV Weeder PD Avruch JH Saeidi N Ozer S. Metabolic profiling during *ex vivo* machine perfusion of the human liver. Sci Rep. (2016) 6:22415. 10.1038/srep22415 26935866 PMC4776101

[93] Goldaracena N Echeverri J Spetzler VN Kaths JM Barbas AS Louis KS. Anti-inflammatory signaling during *ex vivo* liver perfusion improves the preservation of pig liver grafts before transplantation. Liver Transpl. (2016) 22:1573–83. 10.1002/lt.24603 27556578

[94] Yang L Cao H Sun D Hou B Lin L Shen ZY. Bone marrow mesenchymal stem cells combine with normothermic machine perfusion to improve rat donor liver quality-the important role of hepatic microcirculation in donation after circulatory death. Cell Tissue Res. (2020) 381:239–54. 10.1007/s00441-020-03202-z 32347385 PMC7369267

[95] Liu Y Zhang W Cheng Y Miao C Gong J Wang M. Activation of PPARgamma by Curcumin protects mice from ischemia/reperfusion injury induced by orthotopic liver transplantation via modulating polarization of Kupffer cells. Int Immunopharmacol. (2018) 62:270–6. 10.1016/j.intimp.2018.07.013 30036770

[96] Ruan W Liu Q Chen C Li S Xu J. Limb remote ischemic preconditioning attenuates liver ischemia reperfusion injury by activating autophagy via modulating PPAR-gamma pathway. Zhong Nan Da Xue Xue Bao Yi Xue Ban. (2016) 41:918–28. 10.11817/j.issn.1672-7347.2016.09.006 27640790

[97] Chi Y Li J Li N Chen Z Ma L Peng W. FAM3A enhances adipogenesis of 3T3-L1 preadipocytes via activation of ATP-P2 receptor-Akt signaling pathway. Oncotarget. (2017) 8:45862–73. 10.18632/oncotarget.17578 28515350 PMC5542233

[98] Chen Z Wang J Yang W Chen J Meng Y Geng B. FAM3A mediates PPARgamma's protection in liver ischemia-reperfusion injury by activating Akt survival pathway and repressing inflammation and oxidative stress. Oncotarget. (2017) 8:49882–96. 10.18632/oncotarget.17805 28562339 PMC5564815

[99] Lentsch AB Kato A Yoshidome H McMasters KM Edwards MJ. Inflammatory mechanisms and therapeutic strategies for warm hepatic ischemia/reperfusion injury. Hepatology. (2000) 32:169–73. 10.1053/jhep.2000.9323 10915720

[100] Xu X Karreth FA. Pseudogenes as competitive endogenous RNAs: testing miRNA dependency. Methods Mol Biol. (2021) 2324:131–47. 10.1007/978-1-0716-1503-4_9 34165713

[101] Zheng W Men H Li J Xing Y Wu B Wang Z. Global microRNA expression profiling of mouse livers following ischemia-reperfusion injury at different stages. PLoS ONE. (2016) 11:e0148677. 10.1371/journal.pone.0148677 26859886 PMC4747576

[102] Andersson P Gidlof O Braun OO Gotberg M van der Pals J Olde B. Plasma levels of liver-specific miR-122 is massively increased in a porcine cardiogenic shock model and attenuated by hypothermia. Shock. (2012) 37:234–8. 10.1097/SHK.0b013e31823f1811 22089187

[103] Van Caster P Brandenburger T Strahl T Metzger S Bauer I Pannen B. Circulating microRNA-122,−21 and−223 as potential markers of liver injury following warm ischaemia and reperfusion in rats. Mol Med Rep. (2015) 12:3146–50. 10.3892/mmr.2015.3742 25954995

[104] Xiao Q Ye QF Wang W Fu BQ Xia ZP Liu ZZ. Mild hypothermia pretreatment protects hepatocytes against ischemia reperfusion injury *via* down-regulating miR-122 and IGF-1R/AKT pathway. Cryobiology. (2017) 75:100–5. 10.1016/j.cryobiol.2017.01.005 28093198

[105] Mard SA Akbari G Dianat M Mansouri E. Protective effects of crocin and zinc sulfate on hepatic ischemia-reperfusion injury in rats: a comparative experimental model study. Biomed Pharmacother. (2017) 96:48–55. 10.1016/j.biopha.2017.09.123 28963950

[106] Akbari G Mard SA Dianat M Mansouri E. The hepatoprotective and MicroRNAs downregulatory effects of crocin following hepatic ischemia-reperfusion injury in rats. Oxid Med Cell Longev. (2017) 2017:1702967. 10.1155/2017/1702967 28367266 PMC5358472

[107] Wang G Yao J Li Z Zu G Feng D Shan W. miR-34a-5p inhibition alleviates intestinal ischemia/reperfusion-induced reactive oxygen species accumulation and apoptosis *via* activation of SIRT1 signaling. Antioxid Redox Signal. (2016) 24:961–73. 10.1089/ars.2015.6492 26935288

[108] Kim HJ Joe Y Yu JK Chen Y Jeong SO Mani N. Carbon monoxide protects against hepatic ischemia/reperfusion injury by modulating the miR-34a/SIRT1 pathway. Biochim Biophys Acta. (2015) 1852:1550–9. 10.1016/j.bbadis.2015.04.017 25916635

[109] Yu CH Xu CF Li YM. Association of MicroRNA-223 expression with hepatic ischemia/reperfusion injury in mice. Dig Dis Sci. (2009) 54:2362–6. 10.1007/s10620-008-0629-8 19104939

[110] Schueller F Roy S Loosen SH Alder J Koppe C Schneider AT. miR-223 represents a biomarker in acute and chronic liver injury. Clin Sci. (2017) 131:1971–87. 10.1042/CS20170218 28646120

[111] Su S Liu J He K Zhang M Feng C Peng F. Overexpression of the long noncoding RNA TUG1 protects against cold-induced injury of mouse livers by inhibiting apoptosis and inflammation. FEBS J. (2016) 283:1261–74. 10.1111/febs.13660 26785829

[112] Chen Z Luo Y Yang W Ding L Wang J Tu J. Comparison analysis of dysregulated LncRNA profile in mouse plasma and liver after hepatic ischemia/reperfusion injury. PLoS ONE. (2015) 10:e0133462. 10.1371/journal.pone.0133462 26221732 PMC4519261

[113] Chen Z Jia S Li D Cai J Tu J Geng B. Silencing of long noncoding RNA AK139328 attenuates ischemia/reperfusion injury in mouse livers. PLoS ONE. (2013) 8:e80817. 10.1371/journal.pone.0080817 24312245 PMC3842297

[114] Wang J Yang W Chen Z Chen J Meng Y Feng B. Long noncoding RNA lncSHGL recruits hnRNPA1 to suppress hepatic gluconeogenesis and lipogenesis. Diabetes. (2018) 67:581–93. 10.2337/db17-0799 29382663

[115] Tang B Bao N He G Wang J. Long noncoding RNA HOTAIR regulates autophagy *via* the miR-20b-5p/ATG7 axis in hepatic ischemia/reperfusion injury. Gene. (2019) 686:56–62. 10.1016/j.gene.2018.10.059 30367982

[116] Zhang Y Zhang H Zhang Z Li S Jiang W Li X. LncRNA MALAT1 cessation antagonizes hypoxia/reoxygenation injury in hepatocytes by inhibiting apoptosis and inflammation *via* the HMGB1-TLR4 axis. Mol Immunol. (2019) 112:22–9. 10.1016/j.molimm.2019.04.015 31075559

